# Pro-inflammatory effects of DEHP in SGBS-derived adipocytes and THP-1 macrophages

**DOI:** 10.1038/s41598-021-85119-3

**Published:** 2021-04-12

**Authors:** Kristina Schaedlich, Laura-Sophie Beier, Judith Kolbe, Martin Wabitsch, Jana Ernst

**Affiliations:** 1 Department of Anatomy and Cell Biology, Martin Luther University Halle-Wittenberg, Faculty of Medicine, Grosse Steinstrasse 52, 06097 Halle (Saale), Germany; 2 Division of Pediatric Endocrinology and Diabetes Ulm, Department of Pediatrics and Adolescent Medicine, University Medical Center Ulm, Eythstrasse 24, 89075 Ulm, Germany

**Keywords:** Mesenchymal stem cells, Metabolic syndrome

## Abstract

In the member countries of the Organization for Economic Co-operation and Development (OECD), overweight and obesity affect the majority of the population. The use of environmental chemicals, such as the plasticizer DEHP, has largely increased simultaneously with this development. DEHP is an "obesogen" that interferes with normal adipocyte differentiation and energy homeostasis. Obesity in turn is accompanied by chronic low-grade adipose tissue inflammation, leading to metabolic disorders such as type II diabetes. The main actors in adipose tissue inflammation are adipocytes and macrophages. However, the impact of DEHP on adipose tissue inflammation and the crosstalk between adipocytes and macrophages are unknown and the subjects of the current study. The influence of DEHP on inflammation was investigated in human Simpson–Golabi–Behmel syndrome (SGBS)-derived adipocytes and human THP-1 macrophages. The proinflammatory markers IL8, MCP1, IL1β, TNFα and others were measured (qRT-PCR, ELISA) in SGBS-derived adipocytes treated with DEHP [day 0 (d0)–d4; 50 µg/ml] and THP-1 macrophages cultured with conditioned medium (CM) from DEHP-treated adipocytes (SGBS-CM) (from d4 and d8). DEHP exposure led to a proinflammatory state in SGBS-derived adipocytes (e.g., increased secretion of IL8 and MCP1). Surprisingly, exposure of THP-1 macrophages to SGBS-CM did not show DEHP-induced effects. However, we demonstrated that medium containing (pre)adipocyte-secreted factors had a significant impact on the expression and secretion of macrophage and inflammatory markers in THP-1 macrophages in general and led to the significantly increased accumulation of intracellular lipid droplets.

## Introduction

Obesity and its comorbidities, such as type 2 diabetes, are symptoms of metabolic syndrome, a clinical condition that has become a major health issue in not only developed countries. In the twenty-three Organization for Economic Co-operation and Development (OECD) countries, on average, approximately 58% of the adult population is overweight or obese. Furthermore, one-third of children aged 5–9 years living in OECD countries are overweight^1^. According to the World Health Organization (WHO), most of the world’s population lives in countries where overweight and obesity kill more people than underweight. The reasons for this development are the increased intake of energy-dense foods and a decrease in physical activity due to fundamental changes in forms of work and changing modes of transportation^2^. However, over the last 2–3 decades, not only the prevalence of obesity and diseases of metabolic syndrome but also environmental pollution with so-called endocrine disruptors such as di-(2-ethylhexyl)-phthalate (DEHP), TBT, BPA and others have increased ^3^. These endocrine-disrupting chemicals are suspected to act as obesogens on the basis of the Developmental Origins of Health and Disease hypothesis^4–6^. Our research aim was to investigate the influence of early, time-limited and environmentally relevant exposure to DEHP (50 µg/ml) on the development, metabolism and function of differentiating preadipocytes in an invitro human model of Simpson–Golabi–Behmel syndrome (SGBS). The focus of the current is on potentially predetermined dysfunction in cytokine expression and secretion with the establishment of a chronic proinflammatory phenotype in mature adipocytes.

The progression of metabolic dysfunction in obesity is associated with chronic low-grade inflammation characterized by inflammatory cell infiltration and abnormal cytokine production, which predisposes individuals to obesity-related comorbidities such as insulin resistance^7–13^. Inflammation occurs in turn as a reaction to cell damage caused by infection, physical injury or chemicals within certain tissues^14,15^. A number of chemicals are suspected candidates on the list of obesity-causing factors and have been intensively studied. DEHP is one of these compounds, termed obesogens, which additionally act as endocrine-disrupting chemicals. DEHP is used as an additive in plastic products to increase the flexibility of the material. Due to the ubiquitous use of plastic products, this plasticizer can be found in numerous daily use products, in house dust and medical devices^16^. DEHP exposure is especially high during medical interventions with medical tubing to administer blood, plasma and parenteral nutrition, such as in (neonatal) intensive care^17–21^. Furthermore, in blood bags, the levels of detected DEHP were found to range from 1.8 to 83.2 μg/ml^22^. This concentration range was the basis for our investigation. However, depending on the population and subgroup (neonates, patients under intensive medical care, etc.), human exposure to phthalates varies greatly. A further important factor is that measurements are influenced by the applied analytical methods and specimen type (blood, urine, breast milk, etc.). Wittassek and colleagues reviewed this issue in more detail^23^. As a result of the knowledge obtained by basic research and epidemiological studies in recent years, DEHP has been on the Registration, Evaluation, Authorisation and Restriction of Chemicals (REACH) Authorization List (Annex XIV of REACH) of the European Union (EU) since February 2015.

In a recent study with human SGBS-derived adipocytes, we showed that DEHP exposure significantly lowered the degree of adipogenic differentiation. Additionally, lipolytic activity was elevated, accompanied by the decreased uptake and transport of fatty acids into and within the adipocytes. In addition to these metabolic dysfunctions, we detected higher levels of reactive oxygen species (ROS)^24^. The endoplasmic reticulum (ER) is known to be a major source of ROS and may function as a sensor of general metabolic stress, which is translated into inflammatory responses^25^. Due to these results, we aimed to investigate whether DEHP alters the expression of inflammatory markers such as IL8 and MCP1 in adipocytes and whether conditioned medium (CM) from these adipocytes (at two different developmental stages) would influence the expression of macrophage and inflammatory markers in THP-1 macrophages.

To the best of our knowledge, this is the first study to investigate the correlation between DEHP exposure and human adipocyte inflammation as well as the crosstalk between DEHP-treated differentiating human adipocytes and THP-1 macrophages in vitro.

## Results

### DEHP exposure elevated proinflammatory cytokine secretion in SGBS-derived cells

**Figure 1:**
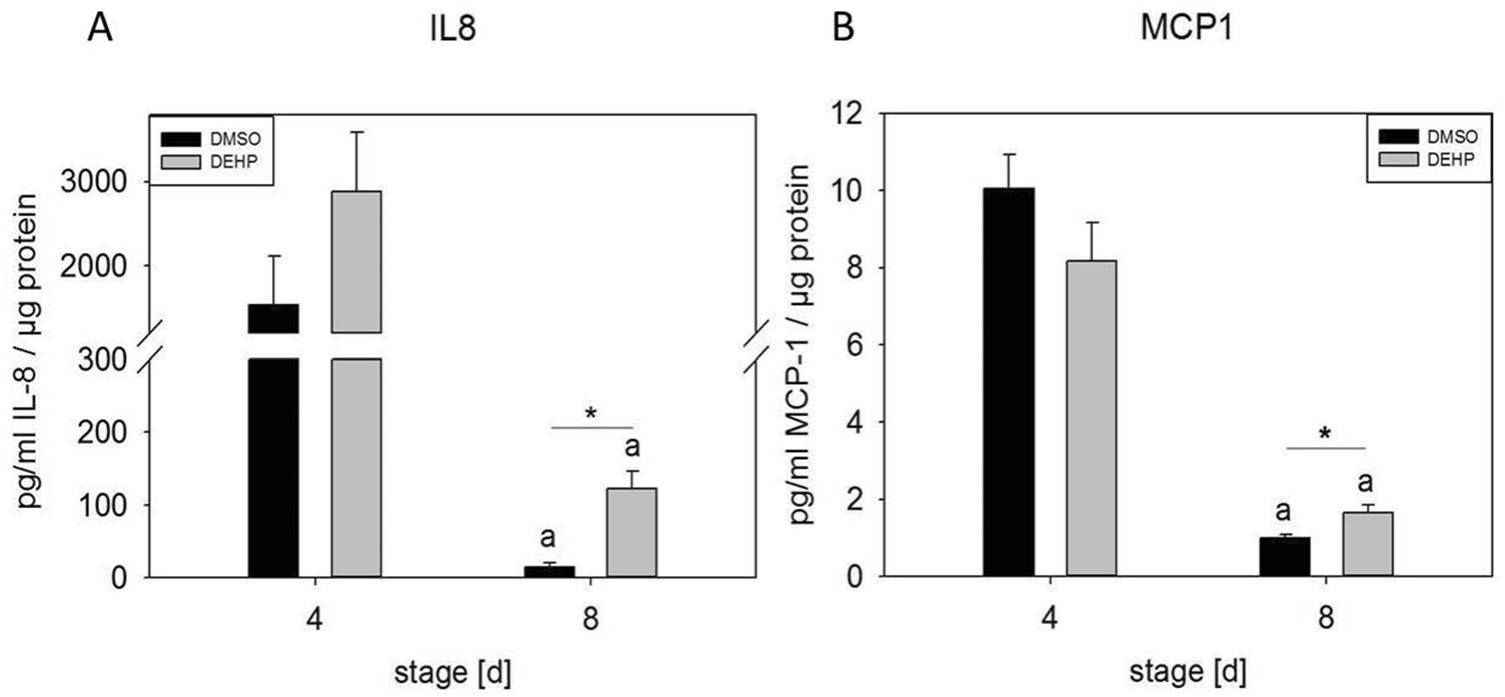
IL8 and MCP1 ELISA with supernatants from SGBS-derived cells. SGBS-derived cells were exposed to DEHP [50 µg/ml] from d0–d4 of adipogenesis. Samples of the cell supernatant were taken at d4 and d8 of differentiation. IL8 and MCP1 ELISA was performed. The data were statistically analyzed by one-way ANOVA on ranks with the Student–Newman–Keuls method. N = 7, n = 1 (4 pooled wells); *p ≤ 0.05 DEHP vs. DMSO at d4 or d8; ^a^p ≤ 0.05 DMSO d4 vs. DMSO d8, DEHP d4 vs. DEHP d8.

Exposure of SGBS-derived cells to DEHP during adipogenic induction from day 0 (d0) to d4 led to an eightfold increase in secreted IL8 and a 1.6-fold increase in MCP1 at d8, supporting the hypothesis that DEHP exposure had a proinflammatory effect on differentiating SGBS-derived cells (Fig. 1). Surprisingly, in the supernatant of DEHP-treated SGBS-derived adipocytes, the secretion of TNFα, resistin and IL1β was not observed with a Human Adipocyte Magnetic Bead Panel (Merck Millipore, Germany) (data not shown). The detection limits were as follows: TNFα [0.1 pg/ml], resistin [2.6 pg/ml] and IL1β [0.5 pg/ml] (data not shown).

### DEHP exposure altered inflammation-related gene expression in SGBS-derived cells

**Figure 2:**
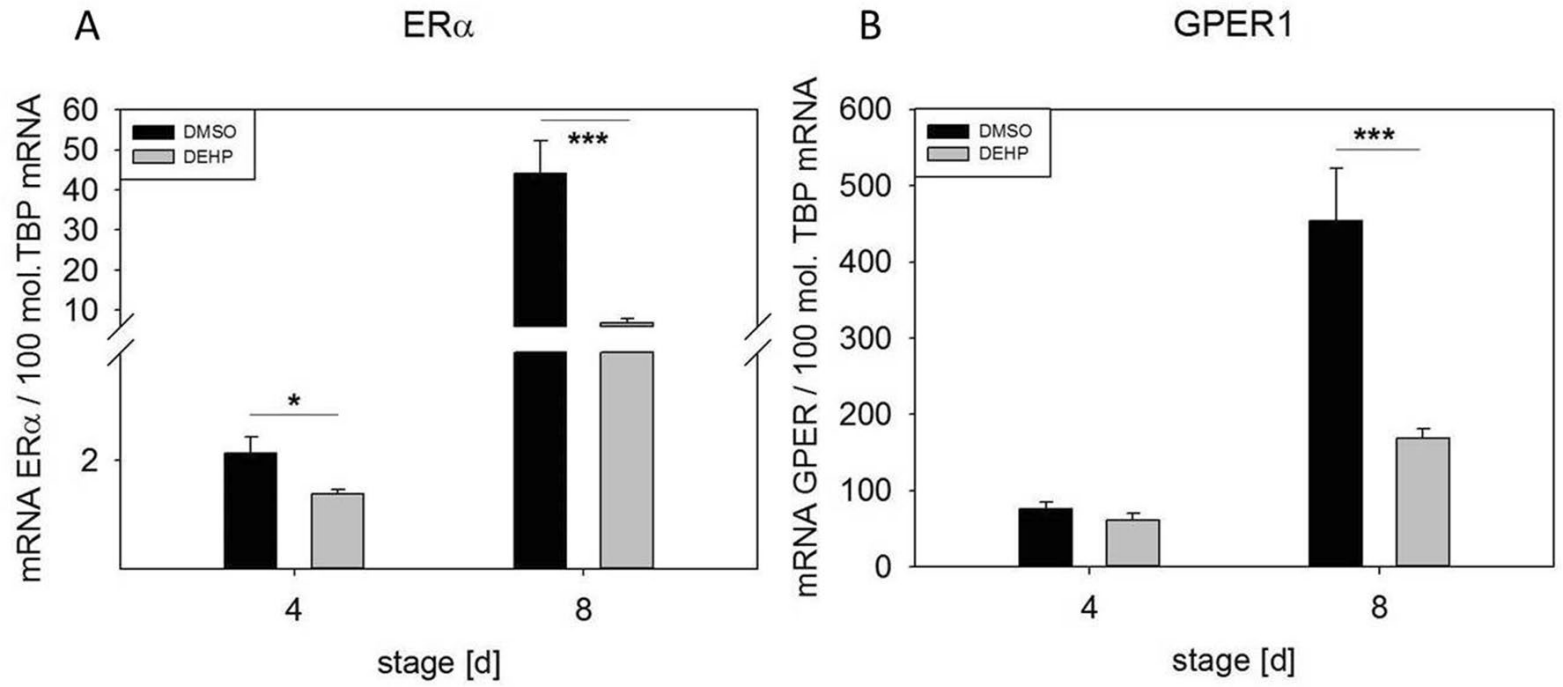
mRNA expression of *ERα* (**A**) and *GPER1* (**B**) in SGBS-derived cells. SGBS-derived cells were exposed to DEHP [50 µg/ml] from d0–d4 of adipogenesis. For qRT-PCR, mRNA samples of (pre)adipocytes were taken at d4 and d8 of differentiation. The TATA-Box binding protein (*TBP*) was used as a housekeeping gene. The data were statistically analyzed by one-way ANOVA. N = 8, n = 1 (4 pooled wells); *p ≤ 0.05; **p ≤ 0.01, ***p ≤ 0.001.

**Figure 3:**
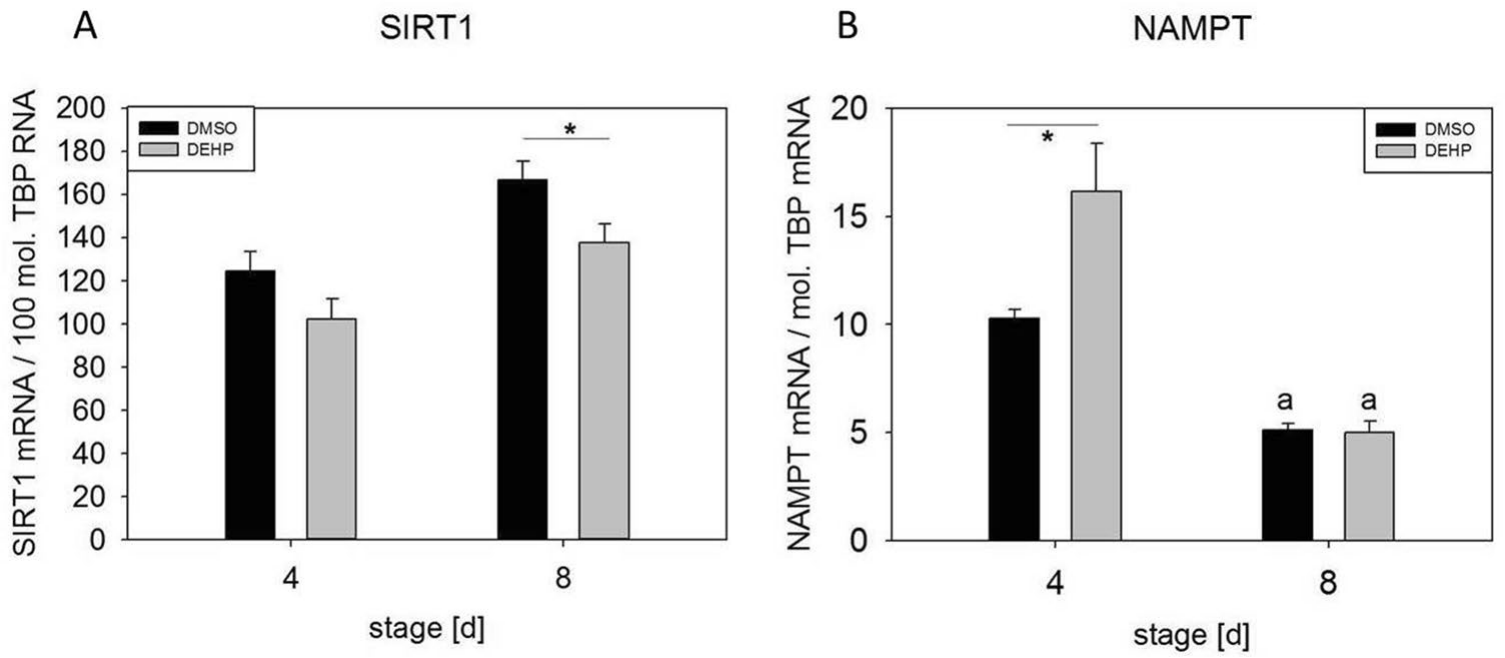
mRNA expression of *SIRT1* (**A**) and *NAMPT* (**B**) in SGBS-derived cells. SGBS-derived cells were exposed to DEHP [50 µg/ml] from d0–d4 of adipogenesis. For qRT-PCR, mRNA samples of (pre)adipocytes were taken at d4 and d8 of differentiation. The TATA-Box binding protein (*TBP*) was used as a housekeeping gene. The data were statistically analyzed by one-way ANOVA on ranks with the Student–Newman–Keuls method. N = 6, n = 1 (4 pooled wells); *p ≤ 0.05 DEHP vs. DMSO at d4 or d8; ^a^p ≤ 0.05.

**Figure 4:**
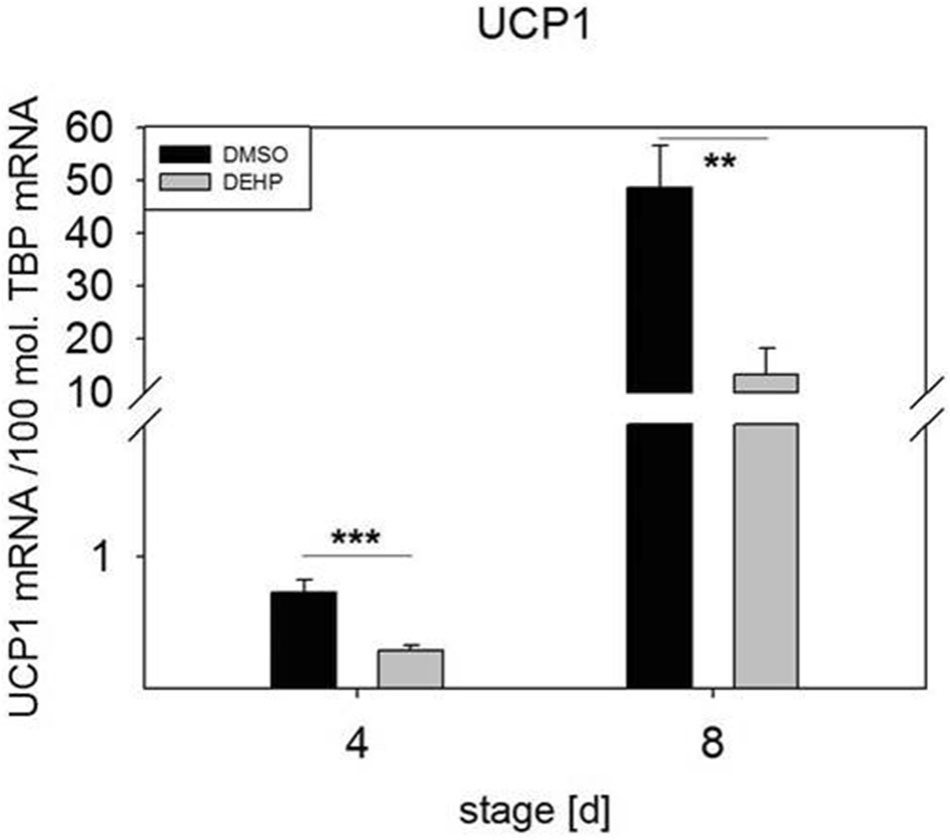
mRNA expression of *UCP1* in SGBS-derived cells. SGBS-derived cells were exposed to DEHP [50 µg/ml] from d0–d4 of adipogenesis. For qRT-PCR, mRNA samples of (pre)adipocytes were taken at d4 and d8 of differentiation. The TATA-Box binding protein (TBP) was used as a housekeeping gene. The data were statistically analyzed by one-way ANOVA. N = 8, n = 1 (4 pooled wells); *p ≤ 0.05; **p ≤ 0.01, ***p ≤ 0.001.

The expression of noncytokine inflammation-related genes after the exposure of SGBS-derived adipocytes to DEHP at d4 and d8 of differentiation was analyzed by qRT-PCR. Expression of the estrogen receptor *ERα* was significantly downregulated at d4 (1.5-fold) and d8 (6.4-fold). The receptor *GPER1* was significantly downregulated at only d8 (2.7-fold) in comparison to its expression in controls (Fig. 2). The downregulation of both receptors is consistent with the suggested proinflammatory environment. Another set of genes of interest were the NAD(+)-dependent histone deacetylase SIRT1 and NAMPT (visfatin), the rate-limiting component of the (NAD+) biosynthesis pathway. Active SIRT1 inhibits NF-κB signaling and plays a role in the resolution of inflammation^25^. *SIRT1* expression was significantly downregulated in DEHP-treated adipocytes at d8 (1.2-fold) (Fig. 3A). However, *NAMPT* expression was significantly upregulated at d4 (1.6-fold) and not changed at d8 (Fig. 3B). Another inflammation-related marker is uncoupling protein (UCP), a specific marker of adipocyte browning. In our study, we observed the significant downregulation of *UCP1* at d4 (2.5-fold) and d8 (3.7-fold) after DEHP treatment (Fig. 4).

### SGBS-CM affected THP-1 macrophage marker expression

**Figure 5:**
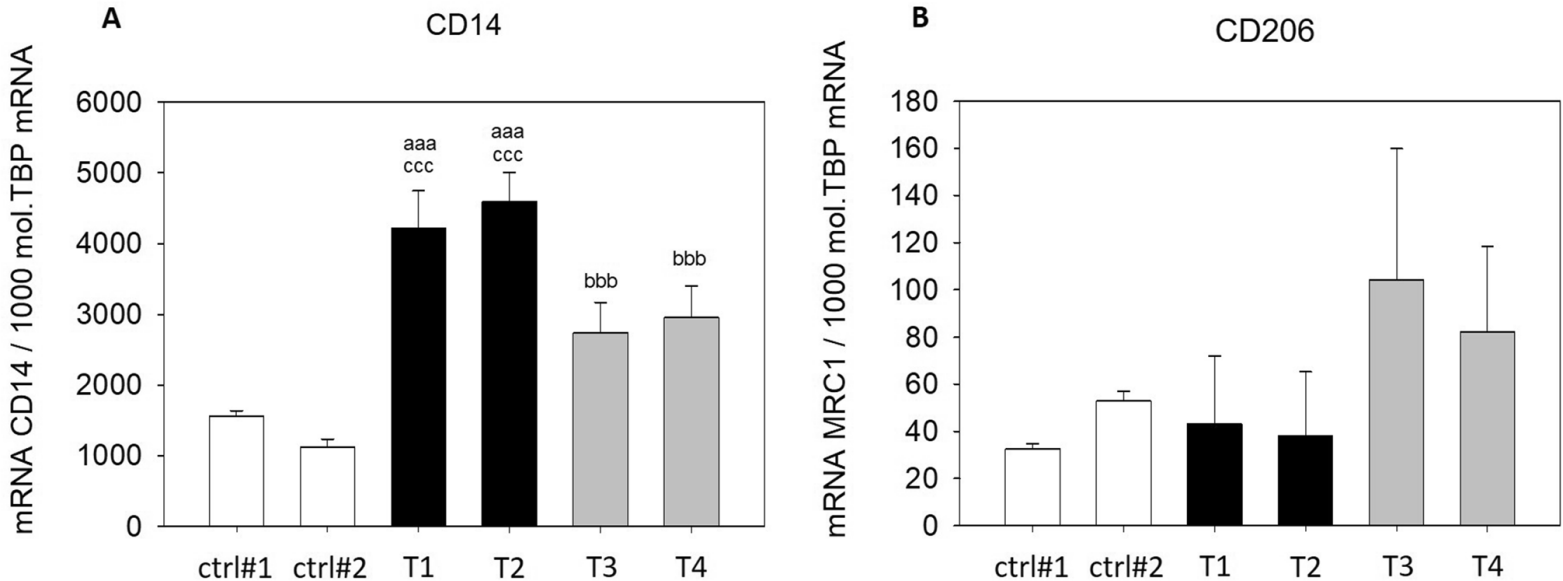
mRNA expression of *CD14* (**A**) and *CD206* (**B**) in THP-1 cells incubated with SGBS-CM. THP-1 cells were incubated for 4 days with SGBS-CM from d4 (T1 or T2) and d8 (T3 or T4) of adipogenesis. The corresponding controls were ctrl#1: RPMI + unconditioned induction medium from d4 (1:1) and ctrl#2: RPMI + unconditioned differentiation medium from d8 (1:1). For qRT-PCR, mRNA samples were from THP-1 macrophages taken at d7 of differentiation. The TATA-Box binding protein (*TBP*) was used as a housekeeping gene. The data were statistically analyzed by one-way ANOVA on ranks with the Student–Newman–Keuls method. N = 6, n = 1 (4 pooled wells); *p < 0.05, DEHP vs. DMSO at d4 or d8; ^a^p < 0.05, ^aa^p < 0.01, ^aaa^p < 0.001, THP-1 + SGBS-CM [d4] vs. ctrl#1 or ctrl#2; ^b^p < 0.05, ^bb^p < 0.01, ^bbb^p < 0.001, THP-1 + SGBS-CM [d4] vs. [d8].

To assess the influence of adipocyte-secreted factors after DEHP exposure, CM from adipogenic differentiated cells on d4 and d8 was added to THP-1 macrophages after PMA treatment. Expression of the macrophage marker genes *CD14* (M1 marker) and *CD206* (M2 marker) was analyzed by qRT-PCR. *CD14* was upregulated after incubation with CM from SGBS-derived cells (SGBS-CM) [d4] and [d8] compared to the level in the corresponding control media from d4 and d8 (T1: SGBS-CM DMSO [d4]: 2.70-fold and T3: SGBS-CM DMSO [d8]: 2.43-fold). Furthermore, the expression of *CD14* significantly decreased from d4 to d8 in SGBS-CM collected under both conditions with (by 36%) and without (by 35%) DEHP treatment (Fig. 5A). The expression of *CD206* did not differ with SGBS-CM (at d4 or d8) and the corresponding control media. It was only upregulated from d4 to d8 under both conditions of SGBS-CM—with (2.16-fold) and without DEHP treatment (2.42-fold) (Fig. 5B). No DEHP-dependent effect was detectable for either macrophage marker gene.

### SGBS-CM promoted the formation of lipid droplets in THP-1 macrophages

**Figure 6:**
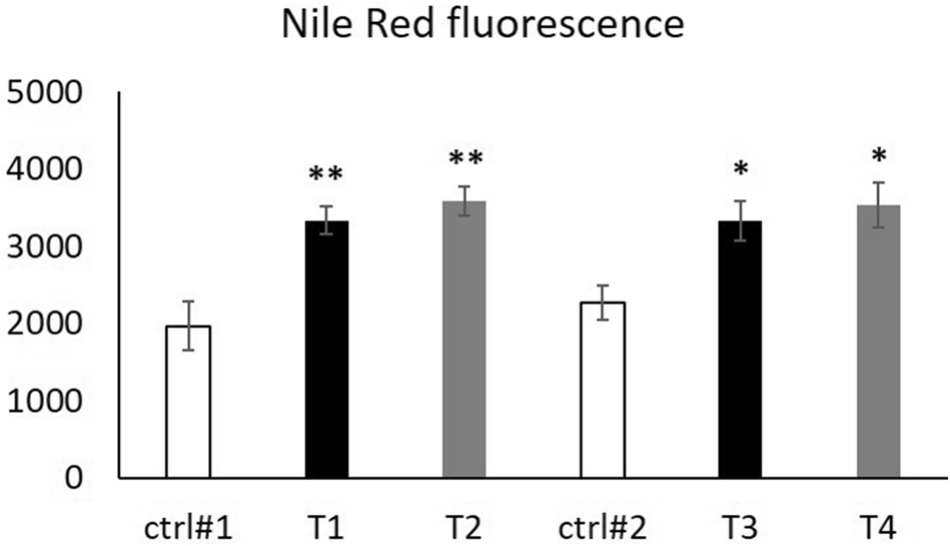
Nile red fluorescence in THP-1 cells incubated with SGBS-CM. THP-1 cells were incubated for 4 days with RPMI:SGBS-CM (1:1) from d4 [containing DMSO (T1) or DEHP (T2)] and d8 [previously treated with DMSO (T3) or DEHP (T4)] of adipogenesis. The corresponding controls were ctrl#1: RPMI + unconditioned induction medium from d4 (1:1) and ctrl#2: RPMI + unconditioned differentiation medium from d8 (1:1). To detect the intracellular lipid content of THP-1 macrophages on d7, the cells were stained with Nile red for fluorescence measurements. The data were statistically analyzed by Student’s *t* test. N = 4, n = 1 (4 pooled wells) *p < 0.05, **p < 0.01; ctrl1# vs. T1 or T2, ctrl#2 vs. T3 or T4.

The accumulation of lipid droplets in immune cells is a known response to infectious and inflammatory diseases and has been linked to emerging metabolic diseases such as diabetes and obesity^26^. Thus, we analyzed lipid accumulation within THP-1 macrophages by Nile Red fluorescence measurements (Fig. 6). THP-1 macrophages were incubated for 4 days with RPMI:SGBS-CM (1:1) from d4 (containing DMSO (T1) or DEHP (T2)) and d8 (previously treated with DMSO (T3) or DEHP (T4)) of adipogenesis. The corresponding controls were as follows: ctrl#1: RPMI + unconditioned induction medium from d4 (1:1) and ctrl#2: RPMI + unconditioned differentiation medium from d8 (1:1). In general, incubation with SGBS-CM significantly elevated the mean Nile Red fluorescence of THP-1 cells compared to that in the corresponding controls (Fig. 6B). This effect was more significant with SGBS-CM from d4 (T1 and T2) than from d8 (T3 and T4). Nevertheless, this effect was independent of (previous) DEHP treatment.

### SGBS-CM elevated IL8 secretion by THP-1 macrophages and decreased MCP1 secretion to a nondetectable level

**Figure 7:**
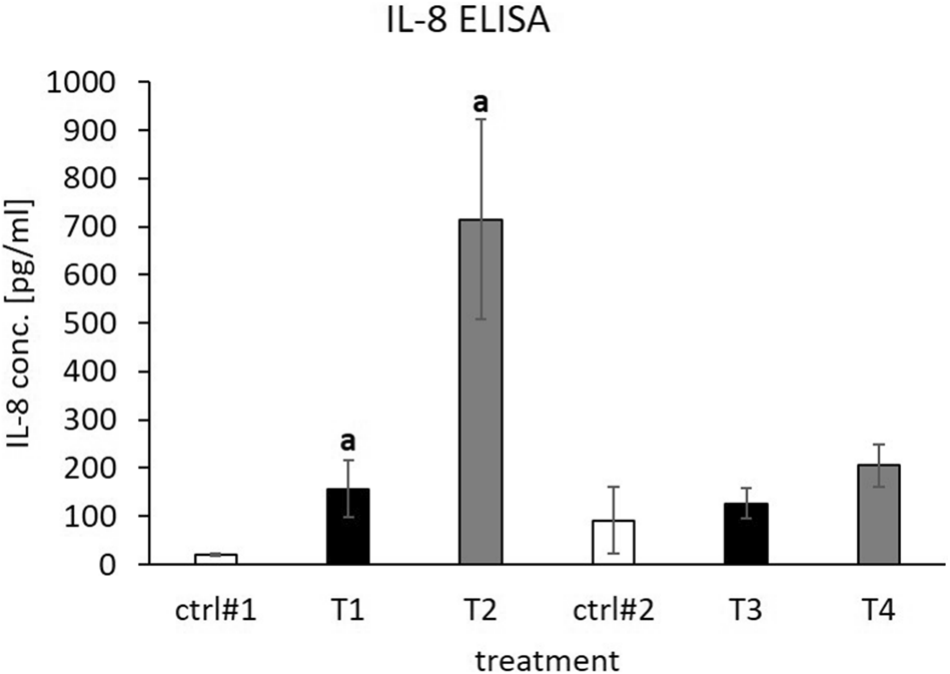
IL8 ELISA with supernatants of THP-1 cells incubated with SGBS-CM. THP-1 cells were incubated for 4 days with RPMI:SGBS-CM (1:1) from d4 [containing DMSO (T1) or DEHP (T2)] and d8 [previously treated with DMSO (T3) or DEHP (T4)] of adipogenesis. The corresponding controls were ctrl#1: RPMI + unconditioned induction medium from d4 (1:1) and ctrl#2: RPMI + unconditioned differentiation medium from d8 (1:1). Samples of the cell supernatant were taken at d7 of differentiation. An IL8 ELISA was performed using the absolute level of both cytokines. The data were statistically analyzed by one-way ANOVA on ranks with the Student–Newman–Keuls method. N = 4, n = 1 (4 pooled wells); ^a^p < 0.05; ctrl#1 vs. T1 or T2.

To investigate whether SGBS-CM from DEHP-treated SGBS-derived cells would have an impact on cytokine secretion in THP-1 macrophages, IL8 and MCP1 ELISAs were conducted. Treatment with SGBS-CM from d4 significantly elevated IL8 secretion by approximately eightfold under treatment 1 conditions (T1: RPMI:SGBS-CM (1:1) of d4 with DMSO) and 36-fold under treatment 2 conditions (T2: RPMI:SGBS-CM (1:1) of d4 with DEHP) compared to those in the corresponding controls [ctrl#1: RPMI + unconditioned induction medium from d4 (1:1)]. The effect of DEHP treatment (T2) seemed to be much stronger than that of treatment with the DMSO vehicle control (T1), but this difference was not statistically significant (p = 0.0771) (Fig. 7). The baseline MCP1 concentrations in the supernatants of the media controls (ctrl#1 and ctrl#2) were relatively low at approximately 3.46 pg/ml. Treatment of THP-1 cells with SGBS-CM led to a decrease in MCP1 in the supernatants of all treated groups to a nondetectable level (T1-T4).

### SGBS-CM altered cytokine- and inflammation-related gene expression

**Figure 8:**
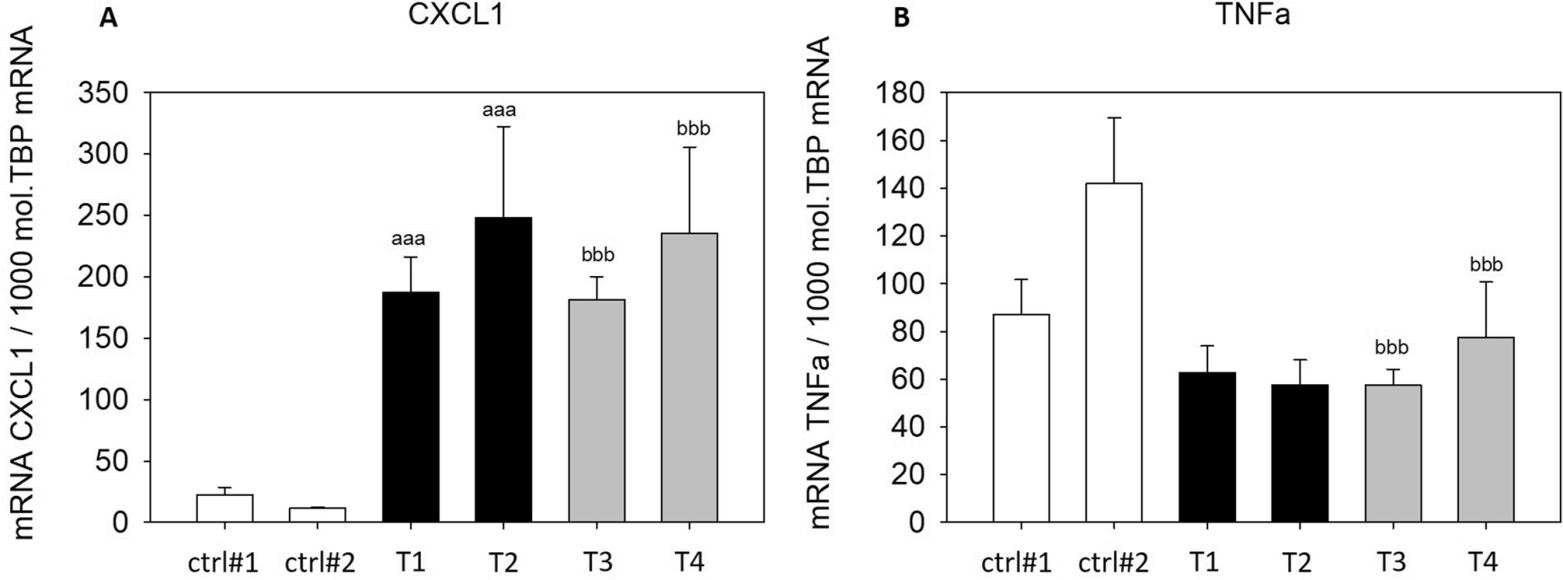
mRNA expression of *CXCL1* (**A**) and *TNFα* (**B**) in THP-1 cells incubated with SGBS-CM. THP-1 cells were incubated for 4 days with SGBS-CM from d4 (T1 or T2) and d8 (T3 or T4) of adipogenesis. The corresponding controls were ctrl#1: RPMI + unconditioned induction medium from d4 (1:1) and ctrl#2: RPMI + unconditioned differentiation medium from d8 (1:1). For qRT-PCR, mRNA samples of THP-1 macrophages were taken at d7 of differentiation. The TATA-Box binding protein (*TBP*) was used as a housekeeping gene. The data were statistically analyzed by one-way ANOVA on ranks with the Student–Newman–Keuls method. N = 6, n = 1 (4 pooled wells); ^a^p < 0.05, ^aa^p < 0.01, ^aaa^p < 0.001, THP-1 + SGBS-CM [d4] vs. ctrl#1 or ctrl#2; ^b^p < 0.05, ^bb^p < 0.01, ^bbb^p < 0.001, THP-1 + SGBS-CM [d4] vs. [d8].

**Figure 9:**
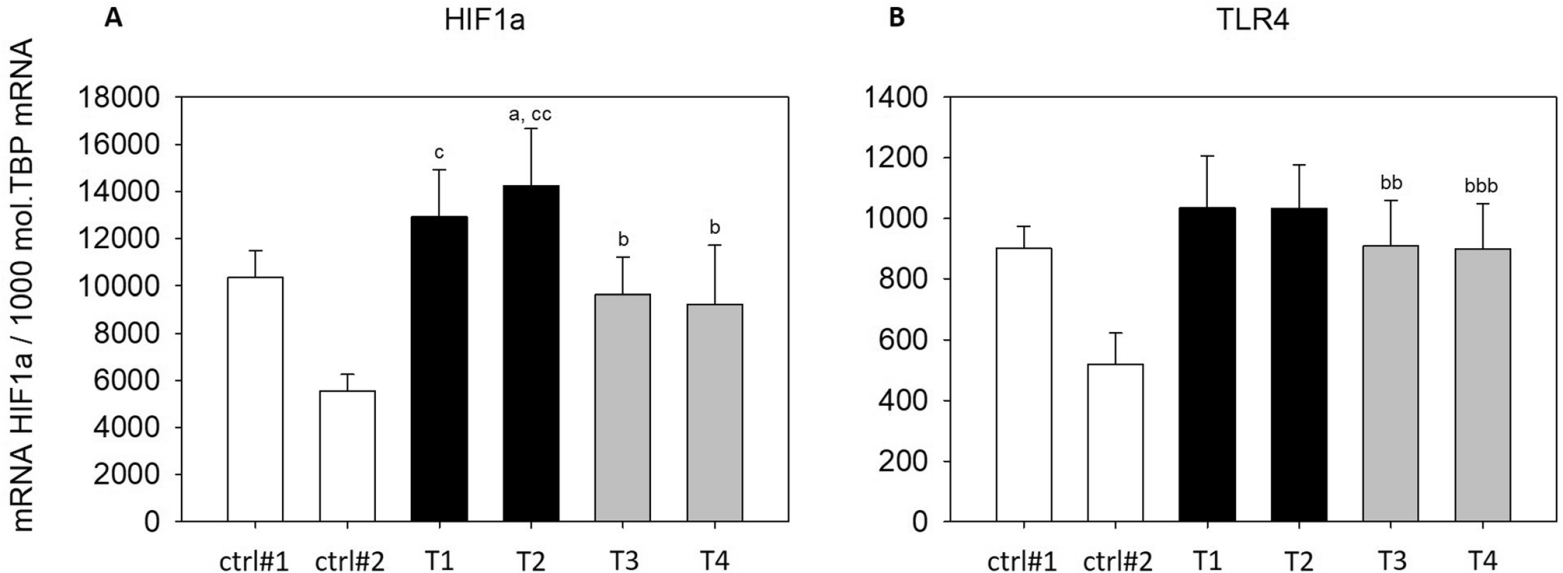
mRNA expression of *HIF1α* (**A**) and *TLR4* (**B**) in THP-1 cells incubated with SGBS-CM. THP-1 cells were incubated for 4 days with SGBS-CM from d4 (T1 or T2) and d8 (T3 or T4) of adipogenesis. The corresponding controls were ctrl#1: RPMI + unconditioned induction medium from d4 (1:1) and ctrl#2: RPMI + unconditioned differentiation medium from d8 (1:1). For qRT-PCR, mRNA samples of THP-1 macrophages were taken at d7 of differentiation. The TATA-Box binding protein (*TBP*) was used as a housekeeping gene. The data were statistically analyzed by one-way ANOVA on ranks with the Student–Newman–Keuls method. N = 6, n = 1 (4 pooled wells); *p < 0.05, DEHP vs. DMSO at d4 or d8; ^a^p < 0.05, ^aa^p < 0.01, ^aaa^p < 0.001, THP-1 + SGBS-CM [d4] vs. ctrl#1 or ctrl#2; ^b^p < 0.05, ^bb^p < 0.01, ^bbb^p < 0.001, THP-1 + SGBS-CM [d4] vs. [d8].

**Figure 10:**
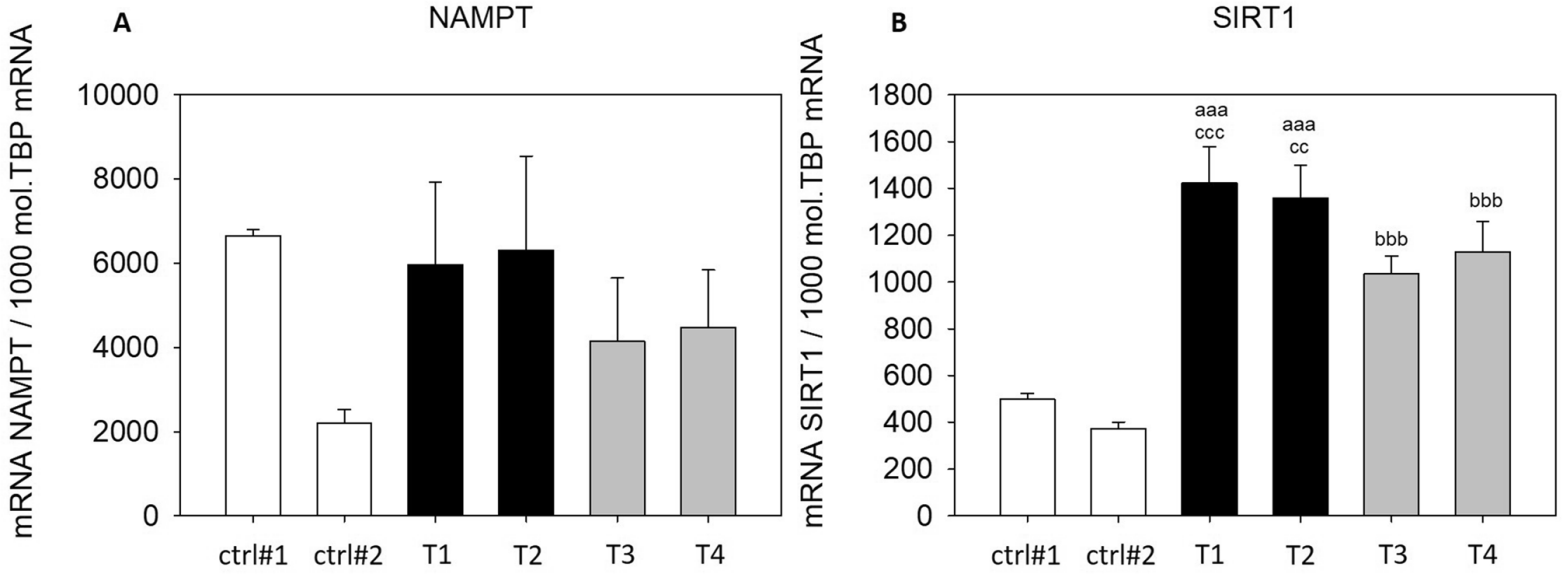
mRNA expression of *NAMPT* (**A**) and *SIRT1* (**B**) in THP-1 cells incubated with SGBS-CM. THP-1 cells were incubated for 4 days with SGBS-CM from d4 (containing DEHP) and d8 of adipogenesis. The corresponding controls were ctrl#1: RPMI + unconditioned induction medium from d4 (1:1) and ctrl#2: RPMI + unconditioned differentiation medium from d8 (1:1). For qRT-PCR, mRNA samples of THP-1 macrophages were taken at d7 of differentiation. The TATA-Box binding protein (*TBP*) was used as a housekeeping gene. The data were statistically analyzed by one-way ANOVA on ranks with the Student–Newman–Keuls method. N = 6, n = 1 (4 pooled wells); *p < 0.05, DEHP vs. DMSO at d4 or d8; ^a^p < 0.05, ^aa^p < 0.01, ^aaa^p < 0.001, THP-1 + SGBS-CM [d4] vs. THP-1 + ctrl#1 or ctrl#2; ^b^p < 0.05, ^bb^p < 0.01, ^bbb^p < 0.001, THP-1 + SGBS-CM [d4] vs. [d8].

In addition to the measurement of secreted IL8 and MCP1, we evaluated the expression of additional cytokines and inflammation-related genes in THP-1 macrophages treated with SGBS-CM. Expression of the proinflammatory cytokine *CXCL1* was significantly upregulated with SGBS-CM ([d4] and [d8]) compared to those in cells treated with the corresponding control media (T1: SGBS-CM DMSO [d4]: 8.44-fold and T3: SGBS-CM DMSO [d8]: 16.18-fold) (Fig. 8A). In contrast, another proinflammatory cytokine, *TNFα,* was significantly downregulated (T1: SGBS-CM DMSO [d4]: by 28% and T3: SGBS-CM DMSO [d8]: by 60%) (Fig. 8B). The levels of both cytokines were unaltered from d4 to d8 (Fig. 8). The NF-κB downstream target *HIF1α* was significantly higher with SGBS-CM [d8] than with the corresponding control medium (T3: SGBS-CM DMSO [d8]: 1.73-fold). Furthermore, from d4 to d8, *HIF1α* was found to be downregulated upon SGBS-CM treatment under both conditions—with (by 35%) and without DEHP treatment (by 26%) (Fig. 9A). Moreover, the expression of *TLR4*, which acts via the NF-κB pathway, was elevated after treatment with SGBS-CM [d8] (1.75-fold) (Fig. 9B). The expression of the inflammation-related genes *NAMPT* and *SIRT1* was also measured. For *NAMPT,* we found its expression to be elevated by only 1.88-fold upon SGBS-CM [d8] treatment compared to treatment with the corresponding control medium (Fig. 10A). The NF-κB antagonist *SIRT1* was significantly upregulated with SGBS-CM ([d4] and [d8]) treatment compared to treatment with the corresponding control media (T1: SGBS-CM DMSO [d4]: 2.86-fold and T3: SGBS-CM DMSO [d8]: 2.77-fold). Incubation with both types of SGBS-CM decreased the expression of SIRT1 (DMSO: 27% and DEHP: 17%) (Fig. 10B). Furthermore, DEHP exposure did not affect expression of the measured cytokine- and inflammation-related genes (Figs. 8, 9, 10).

## Discussion

Chronic inflammation in obese white adipose tissue is a condition that has been observed and demonstrated by many studies in rodents and humans (intensively reviewed in^11,25,27–29^). The factors that trigger adipose tissue inflammation or “metaflammation”, a term proposed by Hotamisligil and colleagues, are not fully understood. One factor may be endoplasmic reticulum (ER) stress, which leads to the accumulation of ROS and the unfolded protein response (UPR), under conditions such as nutritional excess^30–32^. This in turn leads to activation of the NF-κb pathway, resulting in the expression of proinflammatory cytokines^33,34^. To investigate adipose tissue inflammation in vitro, we used SGBS-derived cells and THP-1 macrophages as a human model system, which has been intensively characterized by Keuper et al.^35^. This human model system is a useful and inexpensive tool to gain highly reproducible data. In a recent study, we showed that the exposure of SGBS-derived adipocytes to DEHP led to the significant accumulation of ROS. Furthermore, we detected increased secretion of the proinflammatory adipocytokine leptin, accompanied by decreased secretion of anti-inflammatory adiponectin^24^. In another study, DEHP treatment led to increased concentrations of proinflammatory cytokines in the medium (IL8, TNFα, IL1β and IL6) and to elevated expression of e.g. *CXCL1* in THP-1 macrophages^35^. The mechanism underlying these DEHP-induced effects, however, is not clear. In the current study, we observed significantly elevated levels of secreted IL8 and MCP1 in SGBS-derived adipocytes from d8, and contrary to the previously reported results, no expression of TNFα in SGBS (pre)adipocytes^36^. Furthermore, IL1β was not detectable in cell supernatants, although it was expressed at the mRNA level and significantly elevated at d4 after DEHP treatment (data not shown). According to Maurizi and colleagues, another trigger of “metaflammation” is prolonged overfeeding, leading to adipocyte hypertrophy, which causes the stress response to exceed a certain adipocyte volume threshold^29,37^. However, in our recent study^24^, we observed significantly lower levels of triglycerides and no signs of hypertrophy in DEHP-treated SGBS-derived adipocytes, which nonetheless showed strong evidence of a proinflammatory status.

In addition to classic markers of inflammation, we investigated the inflammation-related markers estrogen receptor alpha (ERα) and G‐protein estrogen receptor 1 (GPER1 or GPR30). A number of studies have identified estrogens as anti-inflammatory modulators of immune function, including their inhibition of IL6 and TNFα^38–40^. Pelikanou and coworkers showed that a splice variant of ERα (ERα36) and GPER1 directly interacted with NF-κB in the nuclei of human monocytes^41^. Notably, SGBS-derived cells also produce and secrete estrogen under standard culture conditions and express *ERα*, *ERβ* and *GPER1* (unpublished data). After DEHP exposure, *ERα* and *GPER1* were significantly downregulated at d4 (ERα) and d8 (*ERα* and *GPER1*) (Fig. 2). Furthermore, the NF-κB antagonist *SIRT1* was also downregulated at d8, accompanied by the downregulation of its downstream target *UCP1,* supporting the proinflammatory profile of DEHP-treated SGBS-derived cells (Fig. 4). These results agree well with a recent study by Lim and coworkers showing that the expression of *UCP1* in adipocytes originating from human was is downregulated by the inflammatory stimuli TNF-alpha and IL-beta^42^. In the current study, we observed the significant downregulation of *UCP1* at d4 (2.5-fold) and d8 (3.7-fold) of adipogenesis after DEHP treatment (Fig. 4). Interestingly, the expression of *NAMPT* was upregulated at d4 and unchanged at d8, suggesting that the decreased *SIRT1* expression observed in our study may not be caused by a lack of available NAD(+).

In the second part of our study, we investigated the effects of SGBS-CM from DEHP-treated SGBS-derived cells on the differentiation of THP-1 macrophages.

At the mRNA expression level, the proinflammatory cytokines *CXCL1* and *TLR4,* which act together with CD14 via the NF-κb pathway^43^, and the NF-κb downstream target *HIF1α* were also significantly upregulated by incubation with SGBS-CM. A recent study by Hörhold et al. characterized the expression profiles of M1- and M2-type macrophages and found *HIF1α* to be especially highly expressed in M1-type macrophages, which supports our findings^44^. These results point towards a proinflammatory status caused by the crosstalk of THP-1 macrophages with secreted (pre)adipocyte factors. Curiously, *TNFα* expression was decreased, and *SIRT1* and *NAMPT* expression was increased. This contradiction may be explained by a study by Liu and coworkers, who observed that chronic lipopolysaccharide (LPS) treatment and the activation of TLR4 increased the expression of *SIRT1* and *NAMPT* and simultaneously suppressed the transcription of *TNF-α* and *IL-1β* in human THP-1 macrophages^45^. Transforming their results to the current study implies that not only chronic LPS stimulation but also chronic exposure to proinflammatory cytokines may increase levels of NAMPT and SIRT1. This hypothesis of course needs to be investigated in more detail. Furthermore, we analyzed secretion of the proinflammatory cytokines IL8 and MCP1 after incubation with SGBS-CM and found IL8 to be significantly increased (up to 36-fold) (Fig. 7) and MCP1 to be decreased to a nondetectable level. Although DEHP (T2) had a strong but not significant effect on IL8 secretion, we are certain that the SGBS-CM induces a proinflammatory reaction by increasing IL8, but not MCP1, secretion in THP1-derived macrophages. This increase in IL8 levels may be caused by the relatively high levels of the proinflammatory adipokine leptin in the SGBS-CM from d4. Cao and colleagues showed a similar effect of leptin-induced IL8 expression in different macrophage models. As we showed before, DEHP exposure increased secretion of the proinflammatory adipocytokine leptin in SGBS-derived adipocytes, which explains its stronger effect on IL8 expression under T2 conditions^24,46^. The decreased MCP1 level was somehow surprising, as it was expected to also increase due to the proinflammatory milieu. A possible explanation for this finding is that, as shown by Gschwandtner and coworkers, MCP-1 is mainly expressed in M2-type macrophages; however, in our model, our cells had an M1 phenotype^47^. Taken together, the results showed no DEHP-dependent effect on THP-1 expression patterns after incubation with either SGBS-CM [d4]—still containing DEHP—or from adipocytes previously treated with DEHP (SGBS-CM [d8]). All of the changes in THP-1 macrophages were caused by the SGBS-CM itself, assuming a basic proinflammatory response to (pre)adipocyte-secreted factors such as leptin and others.

## Material and methods

### SGBS-derived cell culture conditions and DEHP exposure

The human SGBS-derived preadipocyte model used in this study was kindly provided by the laboratory of Professor Martin Wabitsch^48^. After achieving 80% confluence, preadipocytes (d0) were cultured with serum-free induction medium [2 μmol/l rosiglitazone (Cayman #714740), 25 nmol/l dexamethasone (Sigma Aldrich #D-1756), 0.5 mmol/l methylisobutylxanthine (Sigma Aldrich #I-5879), 0.1 μmol/l cortisol (Sigma Aldrich #H-0888), 0.01 mg/ml transferrin (Sigma Aldrich #T-2252), 0.2 nmol/l triiodothyronine (Sigma Aldrich #T-6397), and 20 nmol/l human insulin (Sigma Aldrich #19278)]. On d4, the induction medium was changed to differentiation medium supplemented with 0.1 μmol/l cortisol, 0.01 mg/ml transferrin, 0.2 nmol/l triiodothyronine, and 20 nmol/l human insulin. Exposure of SGBS-derived preadipocytes to DEHP [50 µg/ml] (Sigma Aldrich) occurred from d0–d4 of adipogenic differentiation. DMSO (Sigma Aldrich) was used as the solvent to prepare a 1000-fold stock solution of DEHP, with a maximum concentration of 0.1% DMSO in the culture medium. Additionally, samples in medium containing only 0.1% DMSO were used in each experiment as a vehicle control. At d8 of adipogenic differentiation, SGBS-derived cell culture was finished. The experimental DEHP exposure conditions used herein are comparable to DEHP concentrations used in neonates undergoing medical procedures, such as transfusion or extracorporeal membrane oxygenation, as well as DEHP concentrations found in whole blood and blood components^49,50^.

### Acquisition of SGBS-CM

To obtain SGBS-CM, the cells were cultured and treated as described above. Supernatants at d4 containing DEHP and d8 (without DEHP) were collected. A reasonable amount of CM was obtained by pooling several samples.

### Culture of THP-1 cells with SGBS-CM

To investigate the influence of adipocyte CM (SGBS-CM) with DEHP or DMSO as vehicle on macrophages, THP-1 monocytes were differentiated into macrophages using 100 nM PMA in the CM. THP-1 monocytes were cultured in RPMI medium (Sigma Aldrich, Germany) with 10% FCS (Sigma Aldrich, Germany) and plated on 6-well plates at a density of 2.2 $\times$ 10^4^ cells/cm^2^ before PMA treatment for three days. After PMA treatment, the THP-1 cells became adherent, and the medium was changed daily. The medium was SGBS-CM from d4 (T1: DMSO; T2: DEHP) or d8 (T3: previously treated with DMSO; T4: previously treated with DEHP). At d7, the cells were harvested and processed for RNA and protein analyses. The cell supernatants were collected and stored at − 80 °C for ELISA. The control macrophages were cultured in the same way using unconditioned SGBS induction medium or SGBS differentiation medium + RPMI + 10% FCS (1:1) from d3 to d5 and unconditioned SGBS induction medium or SGBS differentiation medium + RPMI (serum-free) (1:1) from d6 to d7.

### Analysis of THP-1 cell proliferation under DEHP exposure by the BrdU assay

To assure that the THP-1 cells had not been affected by the cytotoxic effects of DEHP exposure by SGBS-CM from d4, a BrdU assay (Cell Proliferation ELISA BrdU, Roche, Germany) was performed. As PMA-induced THP-1 macrophages do not proliferate, THP-1 monocytes were used for this assay. The cells were plated in 96-well plates at a density of 7.4 $\times$ 10^3^ cells/well. They were exposed to DEHP at a concentration of 10 to 50 µg/ml for four days. At d3, BrdU labeling solution (10 µl/well) was added to the cells and incubated for an additional 24 h*.* The reaction was stopped according to the manual, and absorption at 450 nm was measured in a multiwell plate reader (Synergy Mx-Spectrophotometer, BioTek Instruments, Winooski, USA).

### ELISA

The concentrations of the cytokines MCP1 and IL8 in the cell supernatants of adipocytes and macrophages were determined using ELISA kits (MCP1#RAB0054, IL-8#RAB0319, Sigma Aldrich). To calculate cytokine levels in the supernatants of THP1 cells treated with RPMI:SGBS-CM (1:1), the cytokine levels in SGBS-CM before incubation with THP1 cells were subtracted.

### Quantitative real-time PCR (qRT-PCR)

Total RNA was extracted from both cell lines—SGBS and THP-1 cells—using the RNeasy Lipid Tissue Mini Kit (Qiagen). Reverse transcription was performed using RevertAid™ H Minus Reverse Transcriptase (Fermentas, Germany) according to the manual. qRT-PCR was run in an iQ5 Optical System (Bio-Rad Laboratories, Herts, UK) using SYBR Green Mastermix with fluorescein (Eurogentec, Germany). Primer pairs (Table 1) were designed to be exon-spanning, if possible, and tested for specificity by sequencing of the amplification products. The absolute mRNA expression was calculated by serial dilutions of gene-specific plasmid standards. Each assay included cDNA samples in duplicate, the no-template control (NTC) and the gene-specific standard as a positive control. To normalize each sample based on the amount of cDNA, the expression of the housekeeping gene TATA-box binding protein (TBP) was measured. Additionally, a melting curve was determined for all qRT-PCR products to confirm that only a single target had been amplified within the reaction.

**Table 1:** Primers and sequences.

Gene	Specificity	Sequence 5′ → 3'	T_m_ (°C)	Product size (bp)
hu *GPER1*	fw	TTGGCACAGATCCTCGAACAA	60	174
	rv	ATGAAACAGACACCCCAGCTC		
hu *ERα*	fw	CAATGACTATGCTTCAGGCTAC	60	198
	rv	CCACCTTTCATCATTCCCAC		
hu *CD14*	fw	GATTACATAAACTGTCAGAGGC	58	143
	rv	TCCATGGTCGATAAGTCTTC		
hu *CD36*	fw	ACTGAGGACTGCAGTGTAGGA	60	216
	rv	ACAAGCTCTGGTTCTTATTCACA		
hu *CD40*	fw	TATCAAAAAGGTGGCCAAG	60	85
	rv	GAAGATCGTCGGGAAAATTG		
hu *CD206*	fw	AAATTTGAGGGCAGTGAAAG	60	74
	rv	GGATTTGGAGTTTATCTGGTAG		
hu *CXCL1*	fw	AACATGCCAGCCACTGTGAT	62	287
	rv	GCCCCTTTGTTCTAAGCCAG		
hu *HIF1α*	fw	GAAACTACTAGTGCCACATC	60	157
	rv	GGAACTGTAGTTCTTTGACTC		
hu *IL1β*	fw	CATTGCTCAAGTGTCTGAAGC	60	238
	rv	CATGGCCACAACAACTGACG		
hu *IL8*	fw	GGAGCACTCCATAAGGCACAA	60	182
	rv	CAAAACTGCACCTTCACACAGA		
hu *MCP1*	fw	CCGAGAGGCTGAGACTAACC	60	159
	rv	GGGGCATTGATTGCATCTGG		
hu *NAMPT*	fw	CTAATGGCCTTGGGATTAAC	58	114
	rv	TCCAGTGTAACAAAATTCCC		
hu *UCP1*	fw	ACAGCACCTAGTTTAGGAAG	59	160
	rv	CTGTACGCATTATAAGTCCC		
hu *SIRT*1	fw	TTGGCACAGATCCTCGAACAA	62	222
	rv	ATGAAACAGACACCCCAGCTC		
hu *TBP*	fw	TGTGCTCACCCACCAACAAT	60	199
	rv	AGTCGTCTTCCTGAATCCCT		
hu *TLR4*	fw	GGTCAGACGGTGATAGCGAG	60	178
	rv	TTAGGAACCACCTCCACGCA		
hu *TNFα*	fw	AGGCGCTCCCCAAGAAGACA	60	153
	rv	TCCCTGGGGAACTCTTCCCTCT		

### Nile red staining for fluorescence measurements

When the THP-1 cells were ready to be assayed for intracellular triglyceride content, the 12-well plate was removed from the incubator and brought to room temperature. The cell supernatant was removed, and each well was carefully rinsed with 1 ml of phosphate-buffered saline (PBS). Afterwards, each well was filled with 1 ml of room-temperature PBS. A Nile red working solution [5 µg/ml] was freshly made with PBS before use from a stock solution [100 µg/ml] that was stored at -20 °C in the dark. Fifty microliters of the working solution was administered to each well and mixed thoroughly, followed by an incubation period of 10 min in the absence of light. Afterwards, the fluorescence was measured with a fluorimeter with excitation at 485 nm and emission at 572 nm.

### Statistical analyses

Cell culture experiments were performed as at least four independent biological experiments (N ≥ 4). Within one biological experiment, four to six cell culture wells were pooled [N = 1, n = 1 (> 4 pooled wells)]. Data from subsequent analyses by qRT-PCR and western blotting are presented as the mean ± standard error of the mean (SEM). Measurements of cytokine secretion and flow cytometry and Nile red staining experiments were run with at least four independent biological replicates (N ≥ 4) with one individual replicate [n = 1 (≥ 6 pooled wells for mRNA and protein quantification and ELISA)]. All assays were performed with two technical replicates for each sample from which the mean of both measured values was calculated. For statistical analyses, Student’s *t* test or, if necessary, the Mann–Whitney rank sum test, one-way ANOVA or ANOVA on ranks with the Student–Newman–Keuls Method were performed using SigmaPlot software, with automatic testing for normality (Shapiro–Wilk) and equal variance.

## Conclusion

DEHP exposure over 4 days of adipogenesis led to a pro-inflammatory status in SGBS-derived adipocytes. Surprisingly, exposure of THP-1 macrophages to CM from DEHP-treated SGBS-derived (pre)adipocytes, which contained higher levels of the proinflammatory IL8 and MCP1, did not show significant DEHP-induced effects. However, we demonstrated that medium containing (pre)adipocyte-secreted factors such as leptin had a significant impact on the expression of macrophage and inflammatory markers in THP-1 macrophages in general. Furthermore, incubation of THP-1 macrophages with SGBS-CM led to a significantly increase in the accumulation of intracellular lipid droplets.
